# A prospective MRI study of left ventricular iron and function in non-trasfusion-dependent thalassemia intermedia patients treated with desferrioxamine

**DOI:** 10.1186/1532-429X-17-S1-P339

**Published:** 2015-02-03

**Authors:** Antonella Meloni, Mari Giovanna Neri, Chiara Tudisca, Elisabetta Chiodi, Antonino Vallone, Daniele De Marchi, Roberta Renni, Carmelo Fidone, Vincenzo Positano, Alessia Pepe

**Affiliations:** 1 CMR Unit, Fondazione G. Monasterio CNR-Regione Toscana, Pisa, Italy; 2 Dipartimento di Radiologia, Policlinico "Paolo Giaccone", Palermo, Italy; 3 Servizio Radiologia Ospedaliera-Universitaria, Arcispedale "S. Anna", Ferrara, Italy; 4 Istituto di Radiologia, Az. Osp. "Garibaldi" Presidio Ospedaliero Nesima, Catania, Italy; 5 Day Hospital, Ospedale Civile "F. Ferrari", Casarano, Italy; 6 U.O.S. di Microcitemia, Az. Osp. Civile, O.M.P.A. di Ragusa, Ragusa, Italy

## Background

In thalassemia intermedia (TI) patients no observational study prospectively evaluated in the real life the efficacy of the desferrioxamine (DFO) therapy in removing or preventing myocardial iron overload. The efficacy endpoint of this study is represented by the changes in cardiac T2* values and left ventricular (LV) function parameters in non-transfusion dependent (NTD) TI patients after 18 months of desferrioxamine therapy.

## Methods

Among the 325 TI patients enrolled in the MIOT (Myocardial Iron Overload in Thalassemia) network, we selected 129 TI patients NTD. We considered 29 patients who had been received DFO alone between the two MRI scans. Cardiac iron overload was assessed by the multislice multiecho T2* technique. LV function parameters were quantified by cine SSFP sequences. Myocardial fibrosis was evaluated by late gadolinium enhancement (LGE) acquisitions.

## Results

Mean age was 39.69±8.12 years and 14 (48.3%) patients were females. Patients started regular chelation therapy at a mean age of 21.92±15.89 years. The mean administered dosage of DFO via subcutaneous route was 38.46±10.27 mg/kg body weight on 3.32±1.54 days/week. The percentage of patients with excellent/good levels of compliance to the chelation treatment was 82.1%.

At baseline only one patient showed cardiac iron overload (global heart T2*=15.23 ms) but he recovered at the FU (global heart T2*=26.93 ms). All patients without cardiac iron maintained the same status at the follow-up (FU).

Due mainly to technical reasons, LV function was assesses at both baseline and FU MRIs in 24 patients. At baseline all patients had a normal LV ejection fraction (EF) and 4 of them showed a reduced LV ejection fraction (LVEF) at the FU. The changes between the two MRIs in LV volumes and mass indexed by the body surface area as well in EF were not significant.

No movement abnormalities were detected at the baseline MRI while at the FU one patients showed ipokinesia in basal and mid inferoseptal LV wall.

For 21 patients the presence of myocardial fibrosis was investigated at both baseline and FU MRIs, and this subgroup was considered. Three (14.3%) patients had myocardial fibrosis at the baseline, all with a non ischemic pattern.

At the FU two new occurrences of non-ischemic myocardial fibrosis were detected.

## Conclusions

In this small population of sporadically or non transfused TI patients, the DFO therapy showed 100% efficacy in maintaining a normal global heart T2* value but it did not prevent the worsening of the LV function and the occurrence of new myocardial fibrosis.

## Funding

The MIOT project receives "no-profit support" from industrial sponsorships (Chiesi Farmaceutici S.p.A. and ApoPharma Inc.).

